# Factors and pathways involved in capacitation: how are they regulated?

**DOI:** 10.18632/oncotarget.12274

**Published:** 2016-09-27

**Authors:** Shi-Kai Jin, Wan-Xi Yang

**Affiliations:** ^1^ The Sperm Laboratory, College of Life Sciences, Zhejiang University, Hangzhou, China

**Keywords:** capacitation, signaling pathway, calcium Ion, cAMP-PKA, protein phosphorylation

## Abstract

In mammals, fertilization occurs via a comprehensive progression of events. Freshly ejaculated sperm have yet to acquire progressive motility or fertilization ability. They must first undergo a series of biochemical and physiological changes, collectively known as capacitation. Capacitation is a significant prerequisite to fertilization. During the process of capacitation, changes in membrane properties, intracellular ion concentration and the activities of enzymes, together with other protein modifications, induce multiple signaling events and pathways in defined media in vitro or in the female reproductive tract in vivo. These, in turn, stimulate the acrosome reaction and prepare spermatozoa for penetration of the egg zona pellucida prior to fertilization. In the present review, we conclude all mainstream factors and pathways regulate capacitation and highlight their crosstalk. We also summarize the relationship between capacitation and assisted reproductive technology or human disease. In the end, we sum up the open questions and future avenues in this field.

## INTRODUCTION

The phenomenon of mammalian spermatozoa must await a period of time in the female reproductive tract to acquire their fertilizing capacity was first described by Min Chueh Chang and Colin Russell Austin in 1951 in rabbits and rats respectively [1–2], although the specific term ‘capacitation’ was proposed by Austin in 1952 [3]. This is what we called in vivo capacitation now. However, capacitation can also be achieved for spermatozoas in vitro by using particular media containing appropriate compounds and pH [1]. The changes required involve a series of sequential and parallel processes. They begin as soon as the sperm is ejaculated but continue for longer periods in the female tract or capacitation medium. Early research only considered in vivo capacitation while later research has discovered the many common capacitation conditions among different species. In 1977, Iritani et al. demonstrated that boar spermatozoa, which preincubated for several hours in the pig reproductive tract, could penetrate zona-free hamster eggs. This began to indicate that the inducers that occur in oviduct fluid may be similar and common among different species [4].

Capacitation is a prerequirement for fertilization. Only capacitated sperm have the exclusive ability to undergo acrosome reaction and subsequently fertilize the egg. Capacitation occurs, accompanied with other aspects of preparation for the acrosome reaction, at the sperm head whilst preparation for hyperactivation occurs at the sperm tail. Recently, capacitation has been divided into fast and slow events by Visconti et al. [5]. Fast and early events include the activation of the vigorous and asymmetric movement of the flagella. These happen as soon as the sperm leave the epididymis. The slow and late events include changes in the pattern of movement for the sperm and their acquisition of the ability to carry out the acrosome reaction via the stimulation of a physiological agonist and the phosphorylation of tyrosine [5–6]. Despite this fast/slow, early/late split, both processes appear to be regulated by broadly similar molecules (e.g. HCO3-, soluble adenylate cyclase (sAC), cyclic adenosine monophosphate (cAMP)) [5].

**Figure 1:**
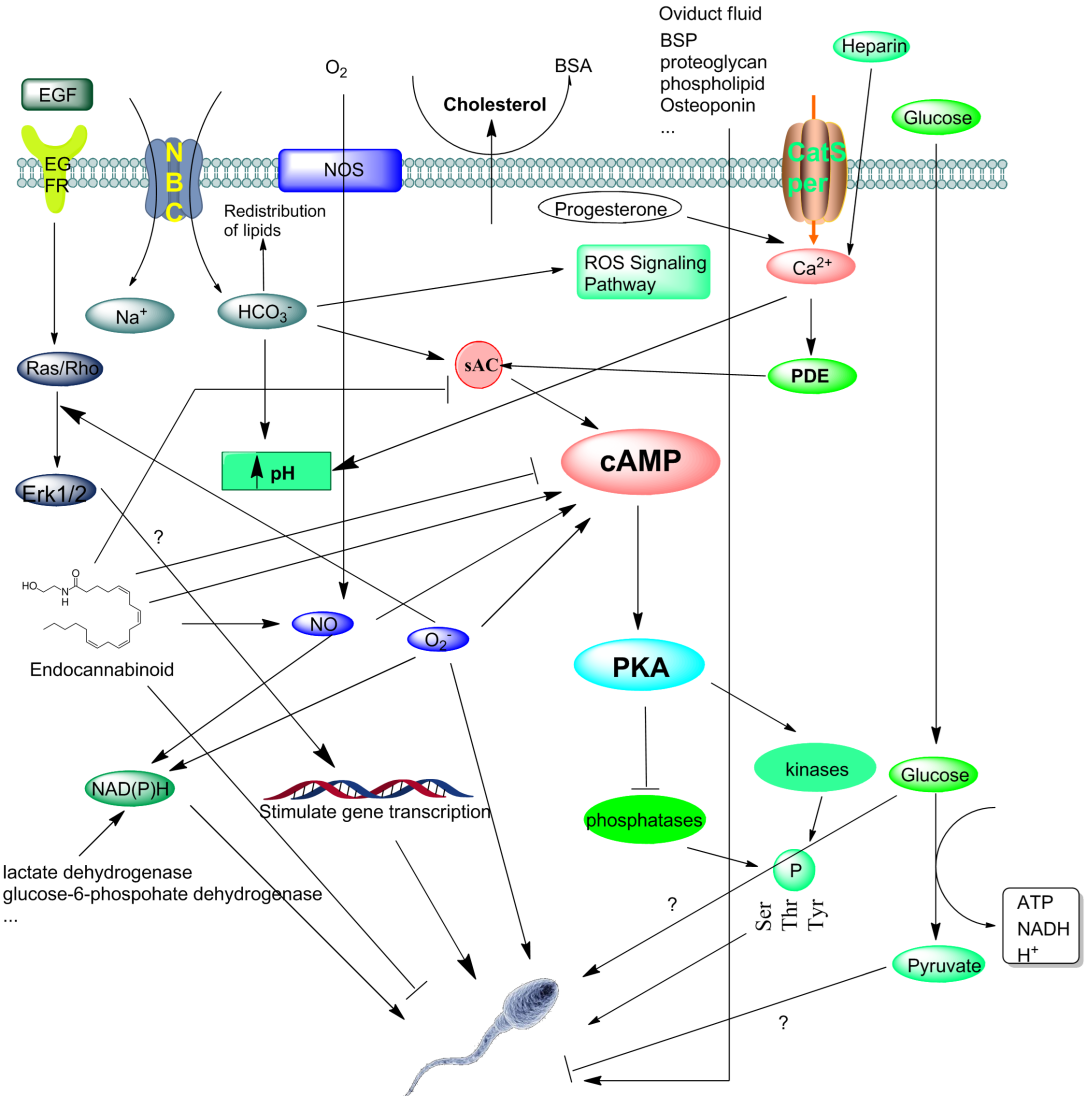
The molecular basis of events associated with capacitation Once ejaculated, removal of cholesterol from the sperm plasma membrane by albumin occurs first. Then the Ca2+ and HCO3- present in the seminal fluid enter the sperm through different channels include Catsper and NBC, respectively. Heparin and progesterone can also help take up Ca2+ into cell, increase intracellular free calcium and pH. The transmembrane movement of HCO3- has been associated with the increase in pHi as observed during capacitation. One of another early happenings during capacitation is the production of ROS, which will trigger and regulate a series of events including protein phosphorylation in a time-dependent fashion. Then spermatoza response to ROS generate the different signaling includes reversible redox signaling and activation of PKA signaling pathway by induce cAMP in a dose-dependent way. Cytosolic and mitochondrial glucose and other lectate are important to supply NADH for the sperm oxidase that produces O2·- for bull sperm capacitation. The factors which activate secondary messenger systems like sAC, then activates PKA. The function of endocannabinoid family to cAMP are controversial, some support it can induce sAC and cAMP production while others hold opposite opinion. PKA through the activation of tyrosine kinases and/or the inhibition of protein phosphatases, then allows an increase in protein phosphorylation and stimulate capacitation or acrosomal exocytosis. Other researches reveal HDL and other substances in the oviduct fluid also induce the capacitation. The function of glucose involved in capacitation remains elusive. Some papers report its absence impair the capacitation while others show glucose per se do not influence capacitation in vitro. EGF stimulates gene transcription during capacitation by activate Ras/Rho, but further mechanisms also remain unknown. The beginning of these events seems to be initiated by the elimination of cholesterol from the sperm membrane. However, all these events are necessary for the sperm to acquire fertilization capacity.

The process of capacitation is under the complicated regulation of various factors (See Figure 1). It is noteworthy that the molecular basis of this process is still not well understood even though more than 50 years have passed since sperm capacitation was first reported. During capacitation, removal of cholesterol from sperm plasma membrane by albumin occurs first. This increases membrane permeability [7–9]. Then a Ca2+ influx occurs through the stimulation of HCO3- [10–13] and membrane channels [14], activates secondary messenger systems including sAC [15–16], finally produce cAMP. The transmembrane movement of HCO3- has been associated with the increase in intracellular pH (pHi) as observed during capacitation. The extracellular signal regulated kinase (ERK) pathway is activated by ligands binding to the membrane receptors and via intracellular activation by reactive oxygen species (ROS). Such activities lead to the phosphorylation of proteins, especially tyrosine. Specifically, a particular type of sAC by acting as intracellular signal [13, 17–18], has been shown to induce tyrosine phosphorylation in stallion sperm [19]. sAC also activated specific protein kinases and phosphates [20–21].

It has been demonstrated that many molecules and proteins, such as steroid hormones in the oviductal or follicular fluids, affect capacitation, the acrosome reaction or hyperactivation [22–27]. Hyperactivation occurs spontaneously and time-dependently with capacitation while the acrosome reaction occurs after the completion of capacitation. Progesterone (P) and estrogen (E) are well-known inducers of capacitation [28–29]. In recent years it has been shown that O2·- (superoxide anion) [30–31], H2O2 (hydrogen peroxide) [32] and NO · (nitric oxide) [33] promote human sperm capacitation. This suggests the oxidative nature of this process. Moreover, some molecules act in a species-dependent manner. c-aminobutyric acid (GABA), for example, acts as an inducer of the acrosome reaction and hyperactivation in humans [27, 34], rams [26], and rats [35], but acts as a suppressor of hyperactivation in hamsters [36].

## IONS, MOLECULES AND REACTIVE OXYGEN SPECIES AFFECT CAPACITATION

### Cholesterol efflux

It is well known that cholesterol efflux has close relationship with capacitation. Many reports indicate that cholesterol efflux causes a decrease of motility, changes in fluidity and alters the lipid distribution in the sperm membrane in order to promote capacitation [37–39]. Withdrawal of cholesterol from the sperm membrane is the initial stage of capacitation with increased fluidity and lower c/p ratio then following. The use of radioactive lipids as molecular probes for monitoring the cholesterol and phosphatidylcholine metabolism revealed that the enzymatic reactions of steroid sulfatase, lecithin, phospholipase A2, and lysophospholipase can provide links between lysophospholipid accumulation in the sperm membranes and the alteration of the c/p ratio [7]. Cholesterol efflux not only supports protein tyrosine prosphorylation (PTP) but also allows phospholipid scrambling that will in turn yield plasma membrane lipid microdomain reorganization promoting sperm-zona pellucida binding and the acrosome reaction [40]. Suzuki et al. used a freeze-fracturing technique, observed that filipin-sterol complexes were initially concentrated in the plasma membrane that overlies the acrosome, but that this concentration decreases dramatically after in vitro capacitation [41].

Cholesterol efflux can activate some transporters, like Na+/HCO3- co-transporter (NBC), or ion channels, such as cation channels of sperm (CatSper), which in turn regulate signaling pathways such as the cAMP-PKA pathway [42]. In addition, the release of cholesterol accompanies sperm capacitation and correlates with an increase in protein phosphorylation [43]. Such crosstalk indicates that cholesterol release and the regulation of phosphorylation pathways are coordinated, occur simultaneously and may result from the same signaling pathway. The mechanism of how cholesterol efflux modulates sperm capacitation is not well understood. Some research has demonstrated that cholesterol concentrates in a specialized domain, known collectively as lipid rafts, on the sperm membrane [44–46] and involved in sorting and distributing lipids and proteins, where they play an important role in signal transduction and in generating cell surface polarity [45].

Bovine serum albumin (BSA) is believed to induce capacitation by functioning as a sink for the depletion of the cholesterol of the sperm plasma membrane and can be replaced by other non-protein cholesterol-binding compounds such as methyl-β-cyclodextrin (MBCD) [47]. BSA can display a dose-dependent function to facilitate cholesterol efflux and capacitation through increasing membrane fluidity in the presence of HCO3- [48]. When hamster spermatozoa were suspended in a medium without BSA, hyperactivation did not occur at all. Thus, it is likely that the removal of cholesterol by albumin is an important signal and an essential trigger for capacitation and hyperactivation [8]. BSA can induce capacitation by generating ROS [49] while BSA-induced sperm capacitation has been correlated with many events that include stimulation of protein tyrosine phosphorylation [17], removal of Zn2+ from cells [50], and the hyperactivation of cell motility [51]. However, there are papers also reported calcium, BSA, bicarbonate, and medium pH have no significant influence on quine sperm cholesterol efflux [52]. Visconti et al. confirmed that the BSA-cholesterol complex could not only inhibit the removal of cholesterol, but also inhibit capacitation and PTP. In addition, cholesterol-binding reagents such as β-cyclodextrins were seen to be capable of activating the signaling pathways involved in the cAMP-PKA pathway [40]. Choi et al. also showed that MBCD treatment for 90 mins removed unesterified cholesterol from mouse sperm which then acted to induce capacitation [53]. Another report showed short-term incubation of MBCD enhance the fertilization rate, may related to the cholesterol efflux [54].

### Intracellular Ca2+ and its channels

Ca2+ regulates sperm capacitation, motility and hyperactivation and triggers the different functional behaviors of sperm that are required for ascending the female tract and fertilizing the oocyte [55–58]. It's not surprising that intracellular Ca2+ in the most center place in capacitation related metabolic network [59]. Also, many ions have relationship with Ca2+ by different ion channels during capacitation (See Table 1). In a manner similar to that in somatic cells, calcium in the sperm increases in two ways. Firstly it is increased via the release of internal stores in the acrosome [60]. Secondly, it increases via the opening of the calcium channel in the membrane [55–56]. There are many works indicate that extracellular Ca2+ enhance capacitation by stimulate multiple ways, however, some works also report Ca2+ negatively regulates PTP in human spermatozoa [61].

**Table 1:** Summary of different ion channels in capacitation

**Name of channel/stimuli**	**Localization on spermatozoa/availability**	**Role in ion flow**	**Role in sperm physiology**	**Effects on capacitation**	**Species**	**Contributors**
CatSper	Principal piece of sperm tail	Ca2+ influx	Ca2+ uptake, hyperactivated motility	induce	human	[69]
Hv1	Principal piece of sperm tail	Ca2+ influx/H+ efflux	induces sperm intracellular alkalinisation	induce	human	[120]
CNG	α subunit is observed along the entire flagellum, whereas the short β subunit is restricted to the principal piece of the flagellum	Ca2+ influx	stimulates influx of calcium via cAMP/cGMP	induce	zebrafish, sea urchin	[98, 102]
GBRC	equatorial segment of the sperm head	Cl- influx	increases capacitation in guinea pig and ejaculated human sperm and ram sperm induces the acrosome reaction in human, mouse and rat sperm	induce	rat	[35]
Kirs	Both tail and head	K+ influx	maintains membrane potential and K+ homeostasis, membrane excitability	induce	mouse	[303]
Slo3	Principal piece of sperm tail	K+ influx	hyperpolarizes sperm membrane, regulates membrane potential; sperm specific	induce	mouse	[304]
Cav	Principal piece of sperm tail	Ca2+ influx	voltage-gated Ca2+ influx into mature sperm cells in response to the application of a high-K+/high-pH extracellular medium	induce	mouse	[72, 81]
Iatp	Midpiece	Ca2+/H+/Na+ influx	energizes mitochondria in the midpiece	induce	mouse	[305]
NBC	/	HCO3-/Na+ influx	induces hyperpolarization through the HCO3- influx; increase protein tyrosine phosphorylation through Na+ influx	induce	mouse	[48]
sNHE	/	Na+ influx/H+ efflux	induces a pHi increase	induce	human, bovine	[306, 307]

Torres-Flores et al. reported that human sperm exposed to the papaverine, one kind of phosphodiesterase inhibitor, displayed the activation of PKA and produced a remarkable increase of the progesterone-induced Ca2+ influx via a cAMP dependent pathway [62]. Although these authors did not evaluate the relationship between in vitro fertility and Ca2+ influx, the changes in pHi and increased tyrosine phosphorylation ultimately may provide a clue relating to sperm fertility competence.

Ca2+ and pHi increase when capacitated spermatozoon encounter the cumulus oophorus [63] or are bound in the glycoproteins of the egg's zona pellucida. This results in the acrosome reaction [14, 64–65]. In the capacitation progress, the most important part is the mechanism that drives the Ca2+ influx in spermatozoa [66]. Previous reports have shown that the mechanisms of Ca2+ influx are controlled by several permeable channel proteins in the spermatozoa [14, 67–68]. However, unlike other cells, spermatozoa do not contain significant amounts of endoplasmic reticulum. Thus, how much Ca2+ is stored in sperm mitochondria remains unexplored. The factors that activate or inhibit the functions of those channels will ultimately help us understand how male fertility is regulated.

Specific ion channels and transporters are located on the sperm plasma membrane. These act to initiate changes in the relevant ions in the sperm cytoplasm [69]. These channels can be regulated by voltage, ligands, pressure or by the depletion of internal stores. The internal stores, principally the endo/sarcoplasmic reticulum, release Ca2+ through channels modulated by Ca2+ itself, or by other second messengers such as IP3, cyclic ADP ribose, nicotinic acid adeninedinucleotide phosphate, or sphingosine 1-phosphate [70]. Hydrolytic enzymes are excreted from the acrosome, especially during the acrosome reaction, to facilitate penetration through the egg's protective vestments [71].

The discovery of a specific calcium channel in the sperm has been a long process. Major types of Ca2+ channels include Cav, CatSper and CNG. The channel responsible for sperm Ca2+ elevation was long been believed to be Cav which, until 2001, was perceived to be the principal Ca2+ conducter of sperm [72–73]. Cav activity is largely intracellular and pH dependent and has long been considered as highly responsive to any change in pH during capacitation [74]. However, calcium influx during in vitro capacitation, as induced by BSA, is due to the activity of the CatSper channel but not Cav [75]. Cav falls into two major functional classes: high voltage- and low voltage-activated channels (HVA and LVA). This function of Cav was supported by electrophysiological identification in testicular spermatocytes (immature spermatogenic cells) using the patch-clamp technique [76–77] and by observation of a putative voltage-gated Ca2+ influx into mature sperm cells. However, most recent report also indicated Cd2+ inhibited the CaV3.1 Ca2+ currents in a concentration-dependent manner, remind us that other ions may also involve in the regulation of sperm [78]. Besides, male mice deficient in Cav2.2, Cav2.3, and Cav3.1 were fertile, indicating that these VGCC channels were not essential for sperm physiology or functioned redundantly [79–81].

In 2001, the first member of a novel family of Ca2+-selective, pH-sensitive ion channels subunits that only expressed in the membrane of the sperm flagellum was discovered. CatSper1 was found to be in control of the entry of positively charged calcium ions into sperm cells and to be required for male fertility [69]. CatSper is essential for sperm hyperactivation and capacitation. Sperm from CatSper-null mice are motile but sterile because they fail to hyperactivate and cannot fully ascend the female tract or penetrate the zona pellucida [69, 82]. Many different psychological stimuli can induce CatSper-dependent increases of intracellular Ca2+. These include pH [67], cyclic nucleotides (e.g., cAMP and cGMP) [16, 83], sAC [83–84], P [85], zona pellucida glycoprotein [86–88] and serum albumin [75]. More specifically, the activity of CatSper is highly increased with intracellular alkalinization [68, 85]. Prostaglandins also activate CatSper channels at very low concentrations (nanomolar) [89–90]. Studies on sperm from humans have shown that mobilization of Ca2+ from a store at the sperm neck region stimulates flagellar activity and can even support hyperactivation in CatSper-null sperm [91–93].

The CNG channel was first proposed by Fesenko in 1985 [94], as functioning to activate light receptors in rod cells by cGMP. CNG channels form heterooligomeric complexes composed of homologous α and β subunits [95]. When heterologously expressed, the α subunits form functional channels of their own. In contrast the β subunits alone are not functionally active. In 1994, Weyand et al. found CNG channels in the mammalian sperm were directly opened by either cAMP or GMP [96]. Their permeablilty to Ca2+ ions make them candidates as the channels for Ca2+ entry into sperm [97], then regulate the motility of sperm and capacitation. Besides, this paper suggests that the downstream cGMP pathway is required in mammalian sperm capacitation and the mechanisms involved include CNG channels and PKG [97]. Furthermore, a recent report showed CNG channel was controlled by pHi, but not cyclic nucleotides in zebra fish, hint us regulation of this channal is complicated [98]. In conclusion, these molecules are undoubtedly highlighted as important therapeutic targets for infertility treatments or potential novel male contraceptives.

Other research reported that the increase of Ca2+ in capacitation may act in a complex manner to control superoxide and nitricoxide formation during the process [99]. It is possible that the initial rise of calcium primeoxidase and NOS activity in sperm induces reactive oxygen species to complete process of capacitation [99].

A recent model proposes another cyclic nucleotides related channels, hyperpolarization-activated and cyclic nucleotide-gated (HCN) channels, which alkalized and depolarized and subsequent lead to the opening of sperm specific CatSper channels [98]. In 1998, one HCN channel has been cloned from sea urchin sperm [100] and 5 years later, the structure of HCN2 channel has been detected [101]. In 2005, a new HCN channel termed SpHCN2 has been found in sea urchin, which has a different motif GFG compare with previous channels, indicating that the ion permeability of this channel may differ from that of its homologues [102]. HCN family is weakly K+ selective and binds to the cAMP and cGMP by its C-terminal fragment [100]. Interestingly, mice and humans deficient in CNG and HCN channels remain fertile and have not been shown to exhibit any defects in sperm function, despite the other related deficiencies in vision and cardiac function [103]. Since CatSper -dependent increase in intracellular Ca2+ concentration can be evoked by adding the cAMP/cGMP [104], cyclic nucleotides may activate CatSper indirectly, possibly via elusive mechanism.

Calcium related pathways are complicated and interrelated during capacitation. One recent research showed that Hsp90 plays an important role in intracellular Ca2+ signaling events in human sperm. Researchers found that geldanamycin, a specific inhibitor of heat shock protein 90, attenuated the rise in Ca2+ in human sperm during capacitation by occupying the ATP binding site of Hsp90, thereby increasing the availability of intracellular ATP [105–106]. Increased ATP levels accelerate Ca2+ efflux via protein kinase C and plasma membrane Ca2+ pump isoform 4, resulting in a decline of Ca2+ in sperm [106–107]. Other reports demonstrated P induced calcium influx during capacitation [85].

### HCO3-dependent regulation in capacitation

After the sperm leave the mammalian epididymis HCO3- plays a key role in their activation. While some early studies have mentioned that the HCO3- content of reproductive fluid likely controls sperm motility and metabolism, they strongly suggested that this is because HCO3- increases the production of cAMP as a messenger that regulates these functions [107]. Many papers have shown that Na+ and HCO3−concentrations are increased while K+ is significantly reduced in the female tract as compared to epididymal fluid [108]. Bicarbonate induces sperm motility and mediates cAMP elevation during capacitation through the stimulation of the activity of sAC, the major adenylyl cyclaseisoform in spermatozoa [109–110]. The activation of PKA then leads to the subsequent increase in the tyrosine phosphorylation of a subset of sperm proteins. Such prototypical pattern of tyrosine phosphorylation represents the best molecularly defined hallmark of capacitation [15, 111].

Several other studies indicate that bicarbonate is the crucial in vitro capacitating agent for several animal species, including the mouse [112] and the bull [113]. The mechanism by which bicarbonate is involved in capacitation involves the induction of sAC. This converts ATP to cAMP [16]. The cyclic nucleotide then dissociates the sperm-specific PKA regulatory subunits from the catalytic subunits [114]. Further study reveals that the phosphorylation of tyrosine is altered in sAC Null Sperm. Sperm shows only a constitutively tyrosine-phosphorylated hexokinase band when incubated under noncapacitating conditions but displays the characteristic pattern of tyrosine phosphorylation during capacitation [115]. In contrast, noncapacitated sAC null sperm shows a different qualitative and quantitative pattern of tyrosine phosphorylation, revealing that, even prior to capacitation, morphologically normal looking sAC null sperm are different from wild-type. Incubating sAC null sperm in capacitating medium does not alter this protein.

A recent study from Boerke et al. demonstrates that porcine and mouse sperm produce oxysterols when incubated in capacitation media supplemented with bicarbonate [116]. This production was act as a ROS-dependent way that is activated by bicarbonate and can be inhibited or blocked by the addition of antioxidants like vitamin A or E or induced in the absence of bicarbonate with pro-oxidants [116].

### pH plays an important role in capacitation

Intracellular alkalinization is required and always occurs before capacitation. The sperm's pH gradually increases as the sperm travel further up the female reproductive tract. Yao shows that, compared with that in pH 7.2 - 8.2 media, the capacitation and movement of sperm in pH 5.2 - 6.2 media decreases significantly [117]. In addition, the acrosome reaction is also pH dependent and needs sperm intracellular alkalinization [67, 118].

**Figure 2:**
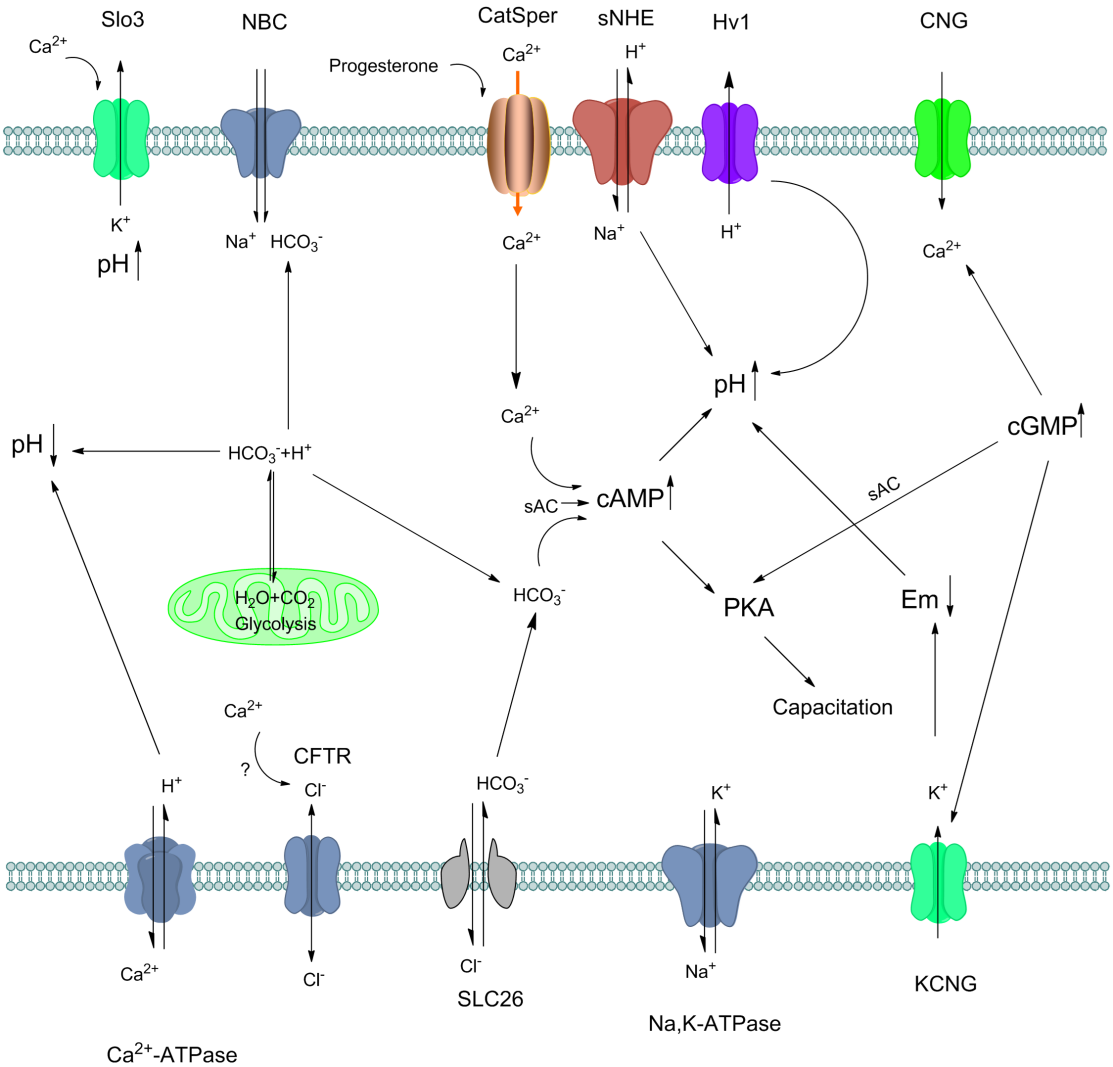
Crosstalk of mammalian sperm pH i and other ions regulation during capacitation. Appropriate sperm intracellular pH is required for sperm capacitation. Protons in sperm may accumulate via different ion exchange, ATP hydrolysis as well as glycolysis. The influx of HCO3- into the sperm is mediated by Na+/HCO3- cotransporters (NBC) and Cl-/HCO3- exchangers (SLC26) so Na+ and Cl- may act in indirect way. Na, K-ATPase alpha 4 is another pump which enhance the sperm motility and hyperactivity during capacitation. Possibly, intracellular Ca2+ may regulate the Cl- exchange, but remain some further confirmation. Ca2+ influx in spermatozoa is principally regulated by CatSper channels. CatSper is regulated by many factors such as progesterone, and its crosstalk with other channels are also intricate. With Ca2+, HCO3- activates sAC, then increase cAMP and leads to PKA and protein tyrosine phosphorylation. Sperm specific Na+/H+ exchanger (sNHE) directly induce the pH increase in sperm accompany with other ion channels include Hv1. On the one hand, rapid proton extrusion is carried out by Hv1 in human sperm, which induce capacitation in indirect manner. On the other hand, Slo3 is activated by intracellular alkalinization and Ca2+ in human sperm, maintaining pHi and contributing to the hyperpolarization that occurs during capacitation. cAMP and cGMP are produced from ATP in mature spermatozoa, induce many channels via G-protein, activate sAC that ultimately induce capacitation. In the mitochondria, glycolysis produce H2O and CO2, which then transfer to HCO3- and H+, maintain the sperm pH hemostasis. Cellular HCO3- are equilibrated by the CO2/HCO3- conversion in mitochondria, thus regulate pH in sperm.

Many ion transporters such as CatSper, Slo3 or HCO3- also regulate the pHi via the influx of ions (As summarized in Figure 2). The elevation or decrease of pHi also activate or inhibit channels. Interestingly, artificial intracellular alkalinization of mouse sperm produced a Ca2+ increase [70]. It was still elusive how pHi and Ca2+ changes influence each other to regulate sperm functions while some evidence showed there were channels sensitive to membrane potential and pHi because artificial intracellular alkalinization add of HCO3- and increase the beat frequency of sperm [119]. Specifically, Voltage-gated proton channel 1 (Hv1) is found in principal piece in sperm and acts to extrude prtons from the flagella. The Hv1 molecule was cloned in 2006 and it seems to lack a classical pore region but, instead, is composed of a voltage sensor domain homologous to the voltage sensor of voltage-gated cation channels [120].

Hv1 channel is activated by membrane potential depolarization, endocannabinoid and alkaline extracellular environment caused by outward H+ gradient across the plasma membrane. Unsaturated fatty acids such as arachidonic acid also enhance the Hv channel activity while Zn2+ potently inhibits it [121]. Extracellular Zn2+ blocks the channel at a nano concentration and the inhibition is dependent on extracellular pH. Upon increasing pH, the inhibition effect of Hv1 by zinc also profoundly enhanced [122–123]. During the journey of female reproductive pathway, the zinc inhibition should be halted by albumin chelation and absorption by uterine and oviductal epithelium in order to finish subsequent reaction [124]. The zinc-binding affinity of albumin might be reduced during conditions of increased free fatty acid mobilization, which may as one possible mechanism to explain this phenomenon.

Strong evidence from Krichok et al. shows that the activity of Hv1 is greatly enhanced in capacitated sperm cells and that this is characterized by many activation kinetics [125]. When spermatozoa were incubated in the same capacitating conditions but in the presence of 1 mM Zn2+, both the increase in Hv1 current and in capacitation were blocked [125]. This situation may be associated with channel phosphorylation, a well-known mechanism for Hv1 up-regulation in somatic cells [216–127]. A big difference between human and mouse sperm is in the Hv1 current which is absent in mouse spermatozoa. With this in mind, it is not surprising that Hv1 knockout mice remain fertile [128].

The regulation of pH is fundamental for sperm function. It is complicated and regulated by a lot of transporters of different ions like CO2, HCO3-, H+, as well as their modulators. Until today, many questions regarding the physiological function of some sperm-specific proteins in pH hemostasis are still unanswered. In this sense, more investigations about this area should be done.

### Steroid hormones - progesterone and estradiol in capacitation

Apart from canonical ions involved in the stimulation or inhibition of capacitation, steroid hormones also has been found involve in the complicated process. The most investigated two, P and E, regulate different parts of capacitation from Ca2+ influx to PTP.

P are usually secreted by cumulus and granulosa cells during ovulation and by the corpus luteum cells in the ovary during the postovulatory phase of the menstrual cycle [129]. Apart from their classical roles in the genomic action of steroid hormones, studies also describe rapid nongenomic effects of these hormones in different cells. Rapid nongenomic effects are too fast to be ascribed to the activation of gene expression and may be mimicked also by steroidal molecules impermeable to the membrane that do not require protein synthesis [22]. The nongenomic model proposes that steroid hormones can also initiate signaling from the exterior through-membrane receptors that transduce those signals to the cell.

The presence of P receptor in the human sperm membrane has been studied by many groups [130]. Hamster sperm hyperactivation was seen to be significantly increased and accelerated by P in a dose-dependent manner via a nongenomic mechanism [8]. Although the acceleration of motility of hyperactivated sperm occurred with 10, 20 and 40 ng/mL P, the most effective concentration was 20 ng/mL [8]. P and GABA increase hyperactivation through the GBRC in rat spermatozoa where GABA itself can induce hyperactivation without the help of GBRC [35–36]. In capacitating human sperm, P induces relevant changes such as the Ca2+ influx which occurs within seconds of adding it while acrosome reaction is observed after few minutes [131]. This suggests that these changes may be triggered by nongenomic models [132]. More recently, Romarowski et al. has simultaneously visualized the rise in [Ca2+]i and the process of exocytosis in response to P and found that only a specific transitory increase in [Ca2+]i, which has its origins in the sperm head, promotes the initiation of acrosome reaction for the first time [133]. Moreover, recent investigations have demonstrated that P activates the sperm-specific, pH-sensitive CatSper Ca2+ channel [85, 134]. P also significantly increased the 80-kDa tyrosine phosphorylation of sperm proteins [8]. In addition, low concentrations of P (10 and 100 nm) induce sperm motility and activate tyrosine kinase while higher concentrations (1-10 μm) are required to induce hyperactivation and acrosome reaction [135]. Interestingly, members of MAPK family such as Erk1/2, p38MAPK, c-Jun N-terminal kinase 1 were find change their phosphorylation status after 10-30 minutes of 5 or 10 μm P [135]. Some research has attempted to ascribe the function of P to the influx of calcium by CatSper. But the progesterone-mediated increase of intracellular Ca2+ was essentially unaffected by pre-treatment with mibefradil and pimozide at concentrations previously shown to prevent increase intracellular Ca2+ in response to zona proteins [136]. This model seems do not support the previous calcium related reports [85], because a significant number of the spermatozoa undergo the acrosome reaction in response to P, even in the presence of mibefradil at concentrations that are known to inhibit CatSper [136]. It revealed that the progesterone-related calcium influx may more complicated than we think before. Moreover, the induction of capacitation by P appears to be related to the promotion of oxygen species generation by sperm [137] and the activation of the MAPK but not to the cAMP/PKA cascade [138].

Another important steroid hormone is E, which is also related to the cholesterol efflux and promotes a rapid influx of Ca2+ from the extracellular medium and an increase in the phosphorylation of tyrosine [139]. The effect of E on human sperm hyperactivation has not, as yet, been the subject of investigation. Interestingly, in hamster sperm, E itself did not affect sperm hyperactivation but suppressed P-enhanced hyperactivation when sperm were exposed to E either concurrent to or before P exposure [140]. However, Francavilla et al. reported that pre-incubation with E did not alter the ability of human sperm to fuse with the oocyte nor did it interfere with the enhancing effect of P on the process [141]. There are also some contradictory reports about whether E induces capacitation or hyperactivation. In addition, some research has hypothesized a role for E in human sperm survival via crosstalk between the PI3K/Akt and ERK pathways [142–143]. In this a direct interaction between ERα with PI3Kand ERβ with AKT was observed [142]. Further connections may indeed exist with different signaling cascades including ER, NOS, PKA and AKT. Up to the present, the evidence presented only suggests a possible modulation by steroids under in vitro capacitation conditions. In conclusion, the participation of P and E in sperm functional regulation when interacting with other molecules in vivo seem to require further consideration.

### ROS and its dose-dependent mechanism in capacitation

Many research have indicated that the production of reactive oxygen species (ROS) are expressed in the early period of capacitation. ROS, mainly the superoxide anion (O2·-) and its dismutation product, hydrogen peroxide (H2O2), and the hydroxyl radical (·OH), from an iron-catalyzed reaction involving H2O2 or O2·-, are typically formed by cells incubated under aerobic conditions while numerous oxygen metabolism produces it [144]. Some but not all are free radicals. ROS are able to diffuse and pass through cell membranes and impair most types of cellular molecules and structures, such as lipids, proteins, and nucleic acids with multiple consequences. As previous part described, ROS influenced almost all parts of capacitation. Claude Gagnon and Eve de Lamirande, firstly demonstrate that one of the hallmarks of capacitation, hyperactivation, is a ROS-dependent process more than 20 years ago [30, 145]. The role of ROS in capacitation is dose-dependent, ROS is required for capacitation as key regulators to promote cholesterol efflux [116], cAMP production [146–147] and PTP [145, 148–150] while excessive concentration are toxic due to the destroy of polyunsaturated fatty acid in sperm surface [151–153], sperm-zona pellucida interaction [154], induce an intrinsic apoptotic-like pathway [155] and resulting in embryo development fail finally [156]. Additionally, peroxidative damage to the sperm plasma membrane caused by oxidative stress can result in an irreparable loss of sperm function and long-term infertility, which engender potential change in newborn [157].

The positive role of ROS in the regulating sperm capacitation has long been observed through ROS-specific scavengers that capacitation can be generated by exogenously O2·- and SOD can prevent such triggered capacitation [30, 145]. Data from de Lamirande et al. shows the concentration of O2·- reaches peak in the 15-25 minutes after capacitated incubation and then decreased slowly in the next 1-2 hours. Besides, addition of SOD 30 min or 60 min after capacitation does not prevent the presence of capacitation. These phenomenon suggest that ROS-induced occurred in the beginning of capacitation but not at later stages and may not be required for the whole incubation period to trigger sperm capacitation [137].

There are many researches demonstrated the potentiation of capacitation by ROS is associated with increased PTP and cAMP pathway [156–159]. A couple of proteins may involve in this process including 116kDa, 105kDa and 81kDa [249, 159]. It is likely that some of them are fibrous sheath proteins related to PKA anchoring protein and its precursor [159–160]. Some reports showed ROS can activate membrane targets in order to trigger the intracellular mechanisms involved in sperm capacitation. One possible target is adenylyl cyclase (AC) because ROS stimulate AC in different cellular systems [161]. Besides, add exogenous O2·- or NO. releasing compounds causes an intracellular increase in cAMP sufficient to support capacitation. This is probably due to stimulation of sperm AC because phosphodiesterase activity is not affected by these treatments [162–163]. ROS may even affected the DNA fragmentation in vitro and sperm function [164], which even associated with a range of adverse clinical outcomes including infertility, miscarriage and diseases in offspring [165].

The function of ROS in capacitation is undoubtedly demonstrated, however, the localization of ROS in sperm is not known, nor is it known whether the action of superoxide anion is dependent or independent of cAMP [18]. The source of the free radicals and oxidants that stimulate capacitation remains elusive. While several groups suggested NADPH may as a candidate because inhibit of NOX5, a NADPH oxidase expressed in sperm, reduces spermatozoa motility and heparin-dependent capacitation was inhibited by all NADPH oxidase inhibitors [166] but there is no definitive evidence confirms that [167]. In equine spermatozoa, capacitation in the presence of ROS generated by the xanthine/xanthine oxidase or NADPH led to a significant increase in live acrosome-reacted spermatozoa and phosphorylation of a subset of proteins of molecular weight 30-200 kDa compared to controls [160]. Nonetheless, other findings demonstrated NADH/NADPH induced capacitation and PTP did not appear to involve extracellular ROS since they were neither prevented by SOD or catalase nor associated with an increased extracellular O2·- by spermatozoa [137]. Besides capacitation, bicarbonate induced ROS appears to be required for oocyte fertilization formation due to causing the sperm surface oxysterol formation and a simultaneous activation of reverse sterol transport from the sperm surface [116].

Another important kind of ROS is Nitric oxide (NO·). Many reports showed the inhibition of ROS prevent the capacitation or acrosome reaction in many different species including mouse [168–169], human [148] and bovine [170]. Furthermore, human [171–172], mouse [169], and bovine [173] spermatozoa are all detected NOS isoforms and there is a sevenfold increase in NOS production in spermatozoa incubated under capacitating conditions [162]. NO also involved in the regulation of PKA/cAMP pathway as a second messenger due to the it steady increase by capacitating spermatozoa and NOS inhibitor decreases human intracellular sperm cAMP concentrations during capacitation [162].

The types of ROS involved in capacitation is still not quite sure. For instance, H2O2 appears to participate in this process, as the addition of exogenous H2O2 induces the capacitation of human [32], boar [174] and equine sperm [160]. Also, previously reports have identified two tyrosine kinases, pp60c-sacroma (Src) [175] and c-ABL [176], that are involved in capacitation and up-regulated by H2O2, confirming a role for this oxidant in sperm function. Interestingly, one paper showed that ROS-generating system in sperm do not affect O2·- [174], but another report H2O2, generated by O2·- dismutation, failed to participate in bovine sperm capacitation [166]. These controversial reports hint us to reveal the pathway related to ROS in-depth.

## DIFFERENT PATHWAYS IN CAPACITATION

### cAMP-PKA pathway

Many reports have confirmed that increased intracellular cyclic adenosine monophosphate (cAMP) levels with the related activation of protein kinase A activity and induction of tyrosine phosphorylation are required for the induction of mammalian sperm capacitation [177–178]. In this, cAMP concentrations are regulated via the modulation of its synthesis by the ACs and/or its degradation via the PDEs. Thus general nonspecific PDE inhibitors such as caffeine or specific PDE inhibitors such as sildenafil can induce capacitation by regulating the PKA/cAMP signaling pathway [178–180]. Although we have detected many cGMP-specific or cAMP-specific PDEs, their contributions to capacitation and penetration into oocytes remains unclear [181–182].

In vertebrate sperm the synthesis of cAMP occurs via two kinds of ACs: tmACs and sACs [183–184]. Based on previous data, the tmACs are known to be regulated by G-protein and forskolin, whereas the sACs iare activated by Ca2+ and bicarbonate [185–186]. Bioinformatic network study also demonstrated that cAMP is produced by the tmAC through high cytoplasmic Ca2+, G proteins and membrane potential, and by the sAC directly activated by HCO3- ions [59]. Sperm cells placed in a capacitating medium show sudden increase in PKA activity which occurs in the first minute of exposure. The levels then decline during the next 15 min and increase again to a peak at 30 min of incubation [187]. In human sperm, an increase in the phosphorylation of PKA substrates during capacitation (80 and 105 kDa) increase as early as 5 min after the beginning of the incubation [188], reach their climax later, 15-30 minutes or 3 hours after incubation based on different mediums and conditions [188–189]. The knockout of PKA and sAC in mice has been demonstrated to result in infertility and the failure of capacitation [190–191].

cAMP interacts with many different signals to regulate capacitation. Some research has reported bicarbonate-dependent activation [192]. It has also been reported that nitric oxide, which interacts with both cAMP and cGMP signaling pathways, induces capacitation in human sperm [193]. It is well known that cAMP is produced as a consequence of a counterbalance between sAC and PDEs and that it stimulates PKA which phosphorylates the serine/threonine (Ser/Thr) protein residues [194]. In the absence of the PKA catalytic subunit (PKA-Cα2) mouse sperm show increase sterility because it cannot fertilize zona-intact eggs. This suggests the involvement of PKA in additional stages of sperm production and maturation [194]. Increasing concentrations of dibutyryl cAMP and PDE inhibitors such as IBMX and caffeine in the media during in vitro culture were accompanied by increasing an intensity of tyrosine phosphorylation in boar sperm [192]. However, the specific mechanism of PKA-mediated PTP remains unknown at the present.

In addition, tyrosine phosphorylation is also induced by the cAMP/PKA-dependent signaling cascades and involved in hyperactivation in response to extracellular Ca2+ [195]. Src family protein tyrosine kinase (SFK) inhibition by SK1606 resulted in a decrease in PKA-mediated phosphorylation. This effect can be reversed using the Ser/Thr phosphatase inhibitor okadaic acid [196].

cAMP may induce capacitation directly through specific proteins. In one report, the lack of a sperm surface protein, the long isoform of beta1,4-galactosyltransferase I, prevented the binding of de-capacitating factors during the epidydimal transit of sperm. This resulted in accelerated capacitation due to the constitutive activation of intracellular pathways such as those drived by calcium and cAMP [197]. Another report showed fibtonectin, a glycoprotein that is present in the oviduct fluid and oviduct epithlium, stimulated human sperm capacitation as mediated through the cAMP/PKA pathways [45].

### Endocannabinoid pathway

Endogenous cannabinoids, also known as endocannabinoids, are a conserved family of endogenous unsaturated fatty acid derivatives that are ligands of the cannabinoid receptor. The main characterized endocannabinoids are anandamide or AEA and 2-AG [197–198]. These form an endocannabinoid system with noladin ether and virohamine [199]. Endocannabinoids served as agonists of specific cannabinoid receptors including CB1R and CB2R receptors as well as ion channels like TRPV1 and T-type Ca2+ channels which located on the membrane of the target cells [200]. Signal-transduction pathways involving AEA include those that stimulate AC [201] together with those that activate mitogen-activated protein kinase [202], modulate intracellular Ca2+ concentrations [203–204] and regulate nitric oxide synthases (NOS) [205].

One important part of endocannabinoid system which has been demonstrated well is its participation in the sperm physiology including capacitation. From one of the earliest report on the effect of AEA on sperm in 1994, which use sea urchin sperm as a model to investigate its influence on acrosome reaction and fertilizing capacity [206], many reports indicate that endocannabinoid signaling is involved in the control of some key reactions in male reproductive system [207–208]. Previous reports indicate that endocannabinoid signaling is implicated in the reproductive system of the male and, attenuates different aspects involved in sperm maturation and the acquisition of fertilizing capacity [209].

The influence of AEA on sperm capacitation may be interpreted as different mechanisms. In some in vitro experiments we concluded that AEA reduce the sperm capacitation, then inhibit acrosome reaction through the activation of CB1R by maintaining the low cAMP level in human spermatozoa [209]. Agonist of CB1 and CB2 also impair spermatogenesis and sperm motility [210].

However, there are also some groups that indicate AEA signaling enhances the capacitation. One report demonstrated that during peri-ovulatory period AEA induce bull sperm capacitation by activate CB1 and TRPV1 receptors, with the help of heparin, suggest effects of AEA may differ in different species [211]. AEA also regulates the sperm release from oviductal epithelia with the participation of NO · [212] and calcium [213]. Another report showed that the effects of AEA on human sperm motility were dependent on the reduction of sperm mitochondrial activity, and that it also inhibits capacitation-induced acrosome reaction [214]. Some recent investigations showed AEA concentration fluctuates during the menstrual cycle in both bull and human while the peak of plasma AEA occurs at ovulation and positively correlates with E and gonadotrophin levels, suggesting that hormones like FSH and LH may be involved in the regulation of AEA levels [215–216].

The percentage of motile spermatozoa recovered from the caput of epididymis has a strong increase comparing to wild-type mice in the absence of CB1 signaling, suggesting a physiological inhibitory regulation of endocannabinoids on sperm motility in the epididymis [217]. However, the effect of AEA on sperm motility can be reversed if incubated within 90 minutes [218]. These findings provide evidence that sperm express functional CB1R, and suggest a possible role for the cannabinoid system in the pathogenesis of some forms of male infertility. Some reports didn't find the express of CB2R in sperm while others report the exist [219].

Interestingly, Gervasi demonstrated that Met-AEA inhibited sperm binding to oviductal cells and induced a significant sperm release at nanomolar concentrations but not at lower concentrations [220]. Talevi and Gualtieri demonstrated similar inhibitory effects of sperm binding in bovine sperm, and the subsequent induction of release triggered by heparin and other sulfated glycoconjugates, known capacitative agents [221].

The endocannabinoids system also influences the fertility. Lewis et al. showed a marked reduction of AEA and 2-AG content in infertile seminal plasma, paralleled by increased degradation-biosynthesis ratios of both substances in sperm from infertile versus fertile men [222]. The mean seminal plasma AEA concentrations were significantly decreased in men with abnormal sperm compared with the normal group [218].

### Extracellular-regulated kinase (ERK) pathway

ERK or MAPK related pathways include other vital pathways in spermatozoa development, capacitation and acrosome reaction [223–224]. ERKs, also known a specific subset of the MAPK family, are a class of serine/threonine kinases which phosphorylate several types of proteins. The upstream cascade of the ERK pathway consists of the adaptorproteins of SHC, GRB2 and SOS and the GTP-binding proteins of the RAS or RHO families [20–21]. The ERK pathway is initiated extracellularly by ligands and intracellularly by reactive oxygen species. This results in the activation of Ras and the sequential phosphorylation and the activation of Raf, MEK, finally activates ERK1 and ERK2 [225–227]. Dramatic increase in the phosphorylation of four proteins SHC1, RAF1, MEK1/2, and ERK1/2 have been examined in mouse spermatozoa [228]. Human sperm capacitation is blocked by inhibitors of any of he elements of the ERK pathway and its upstream modulators including Grb2 to MEK [148, 229]. In boar spermatozoa, ERK1/2 significantly regulates capacitation and RAF1 and MEK1/2 may have some lesser influence through crosstalk with different pathways [225]. In addition to ROS, it could also be that RAF1, MEK1/2, and ERK1/2 influences the sequential events of boar sperm capacitation [225]. ERK1/2 mediates signaling in many cell types. However its role in sperm function is unclear. Jaldety and Breitbart showed that ERK1/2 is highly phosphorylated and activated after a short incubation in mouse sperm under capacitation conditions and that this phosphorylation is reduced after a longer incubation through a MAPK-dependent mechanism but is not influenced by Ca2+ [230]. ROS also activates ERK pathway [99]. Specific inhibitors of the ERK pathway reduce tyrosine phosphorylation of cell surface proteins. This indicates that ERK pathway plays important roles in membrane protein phosphorylation during capacitation [228]. Different growth factors such as EGF, PDGF also activate the ERK pathway in many ways [225]. EGF binds with its receptor leading to the tyrosine phosphorylation of the proteins involved in the PKA and MAPK pathways during capacitation and the acrosome reaction [226]. However, capacitation in the presence of 10 or 100 ng/ml EGF did not alter acrosome status, membrane integrity or motility patterns when evaluated after capacitation in boar spermatozoa [231]. Such a contrast indicates that we still require further understanding of the mechanism of EGF on the ERK pathway.

## PROTEIN PHOSPHORYLATION EVENTS LINKED TO SPERM CAPACITATION

### Protein tyrosine phosphorylation

**Figure 3:**
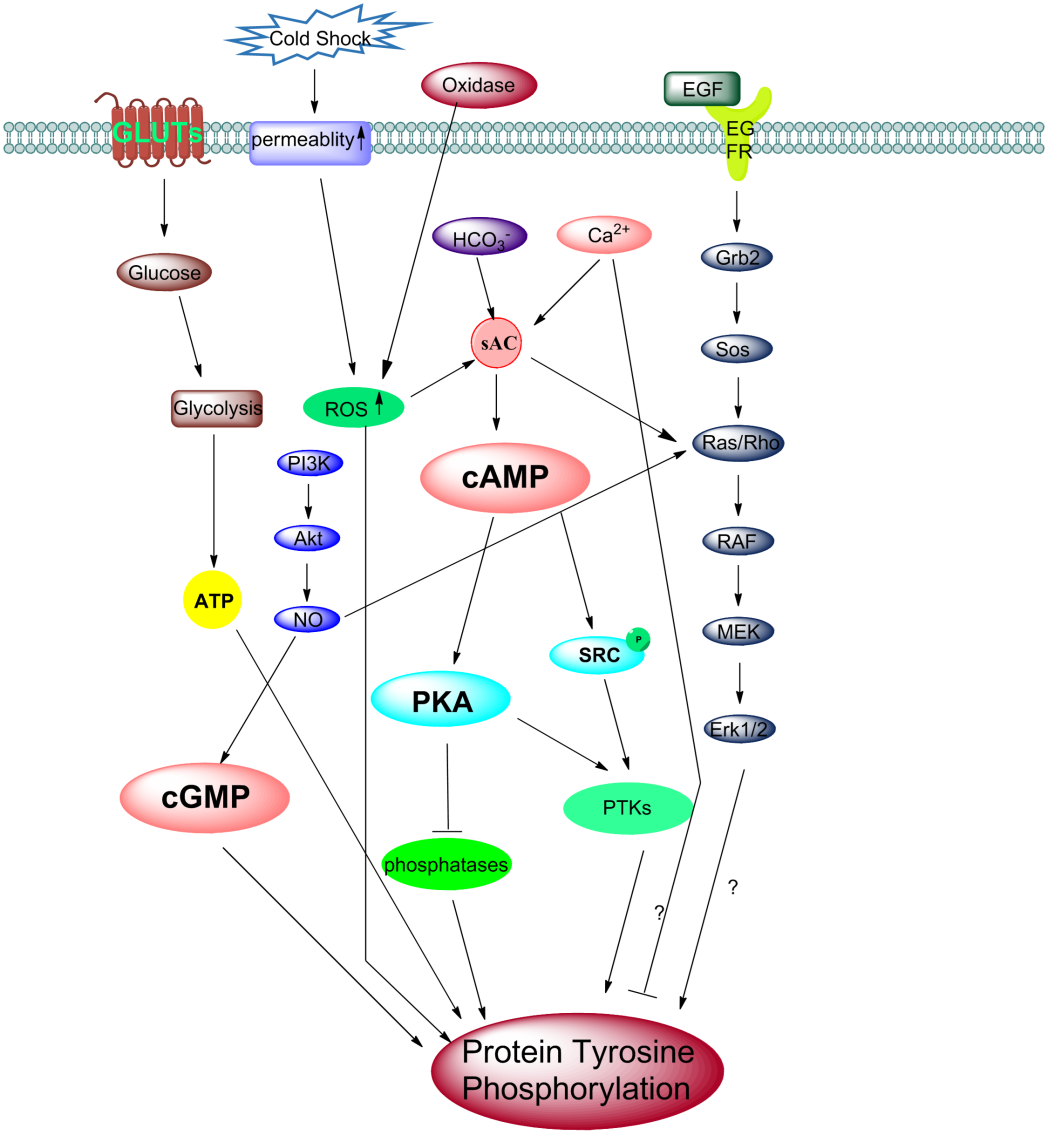
Different models of activation of tyrosine phosphorylation during capacitation A schematic representation of the different molecular models of the activation of tyrosine phosphorylation during sperm capacitation. The removal of cholesterol from the plasma membrane increases membrane fluidity, results in an influx of HCO3- and Ca2+ ions through NBC and calcium channels. The increased intracellular concentration of HCO3-, Ca2+ and ROS, activates the sAC/PKA pathway. However, several papers also report Ca2+ negatively regulates protein tyrosine phosphorylation in human sperm. These phosphorylation of PKA substrates might be directly or indirectly involved in the phosphorylation of MEK-like proteins and subsequently tyrosine residues in fibrous sheath proteins. Also, Src family kinases can inactivate phosphatase, then change the PKA phosphorylation status. Interestingly, cold shock can stimulate and enhance the permeability of the membrane, which also stimulates the ROS pathway and causes an increase in protein tyrosine phosphorylation (PTP). The onset of tyrosine kinases activation is followed by tyrosine phosphorylation. The binding of EGF activates the ERK pathway which increases PTP. NO· can activate the ERK pathway intermediate Ras/Rho protein, while a high NO· can also up act to regulate the cGMP/PKG pathway. The PI3K/Akt axis always modulates the phosphorylation of the Thr-Glu-Tyr motif and tyrosine. It is possible that the downstream effectors such as PDK1 and Akt activate NOS, which then stimulate Ras and the ERK pathway and later cause the increase in PTP. ROS may involve in this pathway as well. Besides, another important factor is glucose, transported by GLUTs into the sperm cell, are useful for ATP generation by glycolysis. ATP is then used for sperm hyperactivation motility and PTP.

Among the post-translational modifications during capacitation, the global upregulation of tyrosine phosphorylation expression has emerged as a critical factor in the regulation of several aspects of sperm function which mediate many important activities in the sperm including capacitation, the acrosome reaction and others (As summarized in Figure 3). Visconti found that in vitro capacitation promotes tyrosine phosphorylation of a subset of proteins between Mr 40,000 and 120,000 [177]. Later, many other findings showed that tyrosine phosphorylation to play important roles during capacitation in multiple species including the mice [177], bovids [232], boars [194, 233], buffaloes [234] and humans [15]. In particular, serum albumin and HCO3-, Ca2+, were seen to be required for tyrosine phosphorylation [235], while the absence of any of these factors prevents both tyrosine phosphorylation and capacitation [177]. During stimulation by these essential components, it has been suggested that 80-kDa tyrosine phosphorylation, is particularly associated with the process [236–237]. Moreover, 80-kDa tyrosine phosphorylation is regulated by Ca2+-calmodulin-dependent signals and protein phosphatase [236]. It has been identified that tyrosine phosphorylated proteins in human sperm, include ion channels, metabolic enzymes and structural proteins which are mainly located in the flagellum [238]. These include CABYR, a calcium binding protein localized in the principal piece of the tail in association with the fibrous sheath [239] and members of the ERK (extracellular-signal regulated kinase) family [240–241]. However, we should also noticed some papers got opposite result as extracellular Ca2+ negatively regulates PTP in human spermatozoa and pHi doesn't drive PTP [61]. Controversial results demonstrated the crosstalk behind every factors seemed more complicated than previous think.

The relationship between protein tyrosine phosphorylation and its regulator indicates a correlation between PTP and cAMP/PKA-induced tyrosine phosphorylation. In this cAMP is a ubiquitous second messenger. The binding of cAMP to the regulatory subunits of PKA allows the dissociation of the tetramer and activation of the catalytic subunits. H89, one kind of PKA inhibitor, decreases tyrosine phosphorylation during capacitation while, by adding analog of cAMP, it can promote the tyrosine phosphorylation in human sperm [47, 138]. In one study, mouse spermatozoa treated with ionophore are able to fertilize without activation of the cAMP/PKA signaling pathway. This indicates that phosphorylation of tyrosine residues might also be associated with calcium influx and upstream pathway [242]. A cAMP-dependent increase of PTP involving PKA in sperm from mice [243], bovids [232], humans [15] and boars [194] has been observed. For example, it was shown that 175k-Da, 93k-Da, 44k-Da, 38k-Da and 20k-Da proteins in boar spermatozoa were phosphorylated in a cAMP-dependent manner [194].

PTPs are activated by of a series of enzymes known as tyrosine kinases (TKs). TKs can be divided into two types, namely receptor tyrosine kinases (RTKs) and non-receptor tyrosinekinases (PTKs). The receptor tyrosine kinases have been identified in sperm of different species such as rabbit, mouse, rat and human [244]. The RTKs are transmembrane proteins which include an extracellular binding domain to a ligand and an intracellular TK domain. However, PTKs such as SRC, FYN are located in the cytoplasm, nucleus, or the inner side of the plasma membrane [245]. The use of specific inhibitors of SRC and/or PKA result in the drastic reduction of tyrosine phosphorylation in capacitated spermatozoa [246–247].

### Protein Ser/Thr phosphorylation

As previously mentioned, capacitation correlates with an increase in PTP as partly mediated by a bicarbonate-dependent cAMP-PKA pathway. As compared with the numerous reports of Tyr phosphorylation, only a few studies have been published relating to the phosphorylation of Ser/Thr residues until now. This reflects the difficulties in performing such studies [248]. Some early research, as early as 1999, described that at least four groups of proteins were determined as being phosphorylated on Ser and that their molecular masses were 43-55 kDa, 94 kDa, 110 kDa and 190 kDa in human sperm, respectively [249]. Some reports have demonstrated proline-directedserine/threonine phosphorylation during capacitation in human sperm [250]. However, the exact role of this pathway remains unknown. Recent studies in mice revealed that a SFK-induced inactivation of serine/threonine phosphatases is also involved in the signaling pathways leading to Tyr phosphorylation [192].

Phosphorylation at the Ser/Thr residues were observed to be increased significantly using calyculin A, one kind of Ser/Thr phosphatase inhibitor [251]. Conversely, Alnagar, using antibodies that recognize the phosphorylation of Ser/Thr residues, noted the dephosphorylation of five phosphorylated proteins in Ser/Thr (96, 90, 64 and 55 kDa) within 15 min of capacitation in the pig [189]. Furthermore, such dephosphorylation did not occur when using calyculin A during sperm capacitation.

Beddu-Addo and his colleagues determined that the Ser/Thr phosphorylation of proteins were dependent on incubation time, the phosphorylation on Ser/Thr being an earlier event than the phosphorylation on Tyr. In addition, these phosphorylation events were reversible and dependent on the presence of BSA and HCO3- [189]. Also, HCO3- induced Ser/Thr phosphorylation was markedly reduced in the absence of BSA in boar sperm [251]. However, Ser/Thr phosphorylation in mouse sperm did not exhibit such a dependence on HCO3- and the role of BSA remains unclear. In addition, based on Visconti's research, PKA activity in capacitating mouse sperm is higher in the presence of BSA than it is in its absence [47]. Furthermore, one recent paper indicated Ser/Thr phosphatases showed different patterns in cryopreserved and freshly ejaculated spermatozoa [252]. Such research indicates that the dynamics of phosphorylation on Ser and Thr residues is very complex and species-specific.

## CAPACITATION RELATED TO ASSISTED REPRODUCTIVE TECHNOLOGY AND FETAL ORIGIN OF ADULT DISEASE

Since Dr. Robert Edwards was awarded the Nobel Prize in 2010 for the development of human in vitro fertilization (IVF) therapy, over four million babies have been conceived using this methodology [42]. In 1978, Steptoe and Edwards reported the birth of Louise Joy Brown, the first successful ‘‘Test-Tube’’ baby [253]. This achievement followed an almost 80 year lack of success of IVF experiments, mainly due to the lack of comprehension relating to capacitation [6]. The elucidation of capacitation led researchers to an awareness that in order to acquire fertilization capacity, mammalian sperm should undergo specific metabolic processes. This realization was followed by the first successful mammalian IVF by Chang in 1959 where capacitated sperm from a black rabbit was transferred to a white rabbit female and resulted in black offspring [254]. Recently, Demyda-Peyrás et al. indicated that the rate of numerical chromosomal abnormalities in IVF early bovine embryos was mainly affected by the concentration of the sperm fertilizing and the sperm capacitation medium [255].

After 1995, another technique called intracytoplasmic sperm injection (ICSI) was developed. Although both belong to assisted reproductive technology, compared with IVF, ICSI greatly reduced the requirements for sperm quantity, motility, and fertilization ability because eggs could now be fertilized not only with fresh sperm but also with sperm that had underwent a frozen-thawed procedure or by sperm of low concentrations or with sperm displaying a lack of motility [256]. The question of whether male factor infertility would affect the outcome of ICSI and the health of the offspring has gained great attention since the introduction of ICSI for the treatment of patients with extremely poor-quality sperm [257–258].

One-sixth couples have difficulty in conceiving and the male factor being the primary cause of infertility in 40% of couples [259]. A major cause of male infertility is spermatogenesis deficiency, including both quality and quantity of the sperm, caused by sperm dysfunction from metabolic deregulation or oxidative stress. As discussed, there are many factors can affect the possibility of capacitation, many of which are strongly related to male spermatogenesis. Absence or change in the genes or proteins which play important roles in development can result in a number of severe systemic diseases [260]. Slc4a2 has been determined to be an expressed specific anion exchange protein which mediates the electroneutral and reversible exchange of Cl- and HCO3- across cell membranes in the male reproductive system, especially in developing spermatozoa and in the epididymal epithelium [261]. Histopathological analysis of Slc4a2-/- testes reveals that spermiogenesis is interrupted resulting in the complete absence of mature spermatozoa and only occasional late spermatids. The mechanism behind this phenomenon may be related to bicarbonate-controlled cAMP signaling dysfunction [261]. Also, CatSper and Slo3 knock-out sperm caused infertility in mice. CatSper-/- mice cannot complete hyperactivation or capacitation. This results in sperm with a loss of motility [69, 262–264]. One recent report even shows malfunction of K+ channels in human spermatozoa might contribute significantly to the occurrence of subfertility in men because they find depolarization of resting membrane potential, were associated with a low rate of fertilization following IVF [267]. In addition, many proteins such as DEFB41 or SPAG11b in the mouse [266–267], or specific molecules such as wortmannin [268], GABA [42] or P [255], influence the process of capacitation. Their lack will also result in sterility.

Another commonly encountered situation among people who exhibits defective spermatozoa related male infertility is failure to bind to the zona pellucida [269]. In a recent report from Bromfield, they postulate that the clinical disruption of sperm-zona pellucida binding may be facilitated by defects in the heat shock protein A2-mediated assembly of zona-recpetor complexes on the sperm surface during capacitation. They found oxidative stress during human sperm capacitation trigger the chemical alkylation of the molecular chaperone heat shock protein A2, a concomitant reduction in surface expression of the zona pellucida-receptor arylsulphatase A and a severe loss of zona pellucida binding ability, which may strongly related to the zona-pellucida binding [154, 270].

In bull IVF experiment, one of the reasons that influence the inconsistent results is the individual effect, known to strongly influence embryo development capacity [271–272], partly because spermatozoa may determine the moment [273] and the duration [274] of the first cleavage. There are many reports studied the relationship between capacitation and embryo development, such as bull sperm which submitted to an oxidative environment negatively influence embryo development when used for in vitro fertilization [156]. Also, DNA damage in the form of strand breaks and base adduct formation is another common feature of defective spermatozoa which may be directly correlated with the normality of early embryonic development [275–276]. Experimental studies have demonstrated that integrity of the sperm DNA and nucleoprotein partly influence mammalian embryo development and implantation, with a threshold of sperm DNA damage beyond which these events are impaired [276–278]. Although the oocyte has the capability to repair damaged paternal DNA, this ability is limited and depends on the grade of damaged DNA [279]. One report showed that melatonin can reduce oxidative damage caused in sperm DNA and improve human sperm quality, which lead to substantial increment in the percentage of good embryos [280]. Nonetheless, such detrimental damage caused by oxidative environment and DNA damage can lead to a predisposition to mutations in the developing embryo with the potential to induce disease in the offspring, not in childhood but also in adult [281].

Unfavorable conditions during life in the uterus and in during childhood not only affects health in childhood, but also increases the potential risk of disease in adulthood. The concept of fetal origins of adult disease (FOAD) was first popularized by Dr. David Barker [282]. The FOAD hypothesis, often called the ‘Barker hypothesis’, states that adverse influences early in development, particularly during intrauterine life, can induce permanent changes in physiology and metabolism which result in increased disease risk in adulthood [283]. After its inception, many studies have provided further evidence for the hypothesis that low birth weight is related to the risk of developing diseases such as coronary artery disease, hypertension, obesity, and insulin resistance in later life. Since it is difficult to retrospectively assess in utero nutrition, birth weight is commonly used as a proxy measurement for that of the nutritional conditions during fetal life.

Although the FOAD hypothesis has been criticized from a number of directions relating to its methodological and theoretical aspects, programming is thus far the most widely accepted theory proposed to underlie such developmental origin/disease links [283–285]. This is the process whereby a stimulus or insult during a sensitive or critical period has irreversible long-term effects on development. In addition to low birth weight, implications of the FOAD include the baby's exposure to stress, both nutritional and nonnutritional, during different critical periods of development which can ultimately result in a disease state. One example in rats showed that the offspring of dams given dexamethasone during pregnancy had reduced birth weight and increased blood pressure and glucose intolerance in adulthood [286–287]. Although a number of papers have suggested that both intrauterine and postnatal growth are important in such factors, it remains unclear exactly which periods are the more important or even whether increased or decreased postnatal growth is harmful [288].

Beyond maternal factors, some reports also show that sperm parameters have relationships with the outcome for the embryo, a factor which seems to be especially vital in ICSI. Large-scale investigations have shown that fertilization rates significantly decrease as semen quality decreases. This indicates that sperm quality can affect the fertilization process [289]. Although researchers failed to observe any statistically significant differences in low birth weight rate among different groups, the high-quality embryo rate (when embryos exhibited 3-4 symmetric blastomeres on the second day of culture and 7-8 symmetric blastomeres on the third day of culture, in the absence of multinucleation and zona pellucida alterations) in the group with normal semen parameters was significantly higher than that in other groups [289].

Francois and his colleagues showed that men with normal semen analysis resulted in offspring with normal birth weight and subfertile men tended to produce offspring of low birth weight [290]. This suggests that bad sperm quality may arouse FOAD. More recent research also suggests strong links between the birth weight and sperm DNA fragmentation or male fertility [291].

Cryopreservation is an efficient way to store spermatozoa. However, cryopreserved sperm showed damage on a variety of ways like decrease in intracellular pH and cAMP and have been deemed to be more sensitive to capacitating agents [292]. In boar sperm, addition of MBCD enhanced sperm vitality [293]. However, in other hand, it hinted this technology may impair sperm capacitation.

The regulation of genes in parents which can pass to their descendant relate to many factors including age or stress. Research from Goriely et al. shows that the mutation levels of FGFR3 and HRAS in sperm increases with paternal age. This revealed a relationship between age and sperm mutations [294]. In addition, Monteleone et al. suggested that prenatal stress exerted strong impacts on fetal brain development and on adult offspring brain function through altering the mRNA expression of GPM6a, a neuronal glycoprotein involved infilopodium extension [295]. La Maestra et al. revealed that living mice exposed to cigarette smoke exhibited significantly increased DNA damage and oxidative stress in sperm cells and those mice showed related morphological abnormalities in their spermatozoa. This, in turn, impaired their sperm function and interfered with sperm fertilization ability [296]. Another meta-analysis presented sufficient evidence in existing literature to suggest that sperm DNA damage has a negative effect on pregnancies that result from IVF and/or ICSI treatment. This hints that there may be a correlative aspect to capacitation.

Until now, the relationship between capacitation and other specific human disease remains unknown. There was no report have tried to connect them directly and exactly. A recent paper confirmed various cancers have deleterious impact on semen quality on large-scale [297]. Although some genes and proteins related to capacitation were reported express in cancer or involved in tumorgenesis [298–300]. A recent paper demonstrated prostasomes, the main sources of cholesterol in seminal fluid and inhibit capacitation [301], can regard as a source of diagnostic biomarkers for prostate cancer [302], reminded us indirect links between capacitation and cancer. Based on aforementioned paragraphs, DNA oxidative damage and ROS-related changes during capacitation should generate scientist's attention for next-step research.

## CONCLUSIONS AND PERSPECTIVES

In view of the notion that capacitation in the female reproductive tract is essential for fertilization, there is a need to unravel the molecular processes involved in this. In recent years, studies have increasingly demonstrated that capacitation plays a pivotal role in the process of fertilization. At the current research level, some questions remain ambiguous and require further investigation. The role of Ser/Thr phosphorylation how it differs from tyrosine phosphorylation, for example, is poorly understood where the related detailed functions and regulations remain to be clarified. Similarly, the effects of many other molecules in the oviduct fluid are still unknown.

Many different models and pathways of capacitation have been mapped and illustrated and it is clear that capacitation is a comprehensive and multistep process. The main aspects elucidated thus far relate to the importance of the cAMP-PKA pathways, which play many important functions, and the aspects of specific protein phosphorylation which may trigger the completion of capacitation. Many components in the in vitro capacitation medium have been confirmed as vital to the process of capacitation. However, in some molecules such as BSA, there seems to be disparate functions in different species. Furthermore, the types of ion channels active in the process also show some minor differences between humans, mice and other animals, which suggest their differing roles, at least to a minor extent, in inducing capacitation.

Despite the abundant research efforts which have been conducted in the exploration of different pathways in capacitation, the current understanding is far from sufficient or complete. The following four aspects, in particular, require further examination. Firstly, although we have illustrated the key aspects of different ion channels with their affiliated pathways, the interrelation of the PKA pathway and PTP pathway is still not clearly understood. Secondly, the ability of the female tract to control the speed of capacitation and the delivery of capacitated sperm to the site of fertilization remains to be investigated. Thirdly, most of research thus far has been conducted in vitro where the action of some agonists and antagonists remain quite different from that in the physiological environment in vivo. For some widespread substances, such as glucose and albumin, their relationships and mechanisms with capacitation are also still not well understood. In the end, how many kinds of kinases and phosphatases are responsible for modulating capacitation and how many ion channels are responsible for the ion fluxes during capacitation remains unanswered. The answer of these questions may be steadily achieved with the advances in proteomic and electrophysiological tools. Addressing such questions will considerably aid the field of infertility treatment, diagnosis and shed much light upon the process of capacitation.
